# Protein based molecular markers provide reliable means to understand prokaryotic phylogeny and support Darwinian mode of evolution

**DOI:** 10.3389/fcimb.2012.00098

**Published:** 2012-07-26

**Authors:** Vaibhav Bhandari, Hafiz S. Naushad, Radhey S. Gupta

**Affiliations:** Department of Biochemistry and Biomedical Sciences, McMaster UniversityHamilton, ON, Canada

**Keywords:** conserved indels, signature proteins, phylogenetic trees, lateral gene transfers, Thermotogae, Archaea, Crenarchaeota, RpoB signatures

## Abstract

The analyses of genome sequences have led to the proposal that lateral gene transfers (LGTs) among prokaryotes are so widespread that they disguise the interrelationships among these organisms. This has led to questioning of whether the Darwinian model of evolution is applicable to prokaryotic organisms. In this review, we discuss the usefulness of taxon-specific molecular markers such as conserved signature indels (CSIs) and conserved signature proteins (CSPs) for understanding the evolutionary relationships among prokaryotes and to assess the influence of LGTs on prokaryotic evolution. The analyses of genomic sequences have identified large numbers of CSIs and CSPs that are unique properties of different groups of prokaryotes ranging from phylum to genus levels. The species distribution patterns of these molecular signatures strongly support a tree-like vertical inheritance of the genes containing these molecular signatures that is consistent with phylogenetic trees. Recent detailed studies in this regard on the Thermotogae and Archaea, which are reviewed here, have identified large numbers of CSIs and CSPs that are specific for the species from these two taxa and a number of their major clades. The genetic changes responsible for these CSIs (and CSPs) initially likely occurred in the common ancestors of these taxa and then vertically transferred to various descendants. Although some CSIs and CSPs in unrelated groups of prokaryotes were identified, their small numbers and random occurrence has no apparent influence on the consistent tree-like branching pattern emerging from other markers. These results provide evidence that although LGT is an important evolutionary force, it does not mask the tree-like branching pattern of prokaryotes or understanding of their evolutionary relationships. The identified CSIs and CSPs also provide novel and highly specific means for identification of different groups of microbes and for taxonomical and biochemical studies.

## Introduction

The understanding of prokaryotic relationships is one of the most important goals of evolutionary sciences. These relationships have been difficult to understand due to the simplicity and antiquity of prokaryotic organisms and disagreements in viewpoints among evolutionary biologists regarding the importance of different factors when grouping prokaryotes. Although earlier studies in this regard were based on morphology or physiology (Cowan, 1965; Buchanan and Gibbons, 1974; Stanier et al., 1976), the field itself has evolved to account for new information brought about by technological or informational breakthroughs, viz. molecular data, DNA hybridization and 16S rRNA (Zuckerkandl and Pauling, 1965; Woese and Fox, 1977; Woese, 1987). The most recent breakthrough involves rapid and easily available sequencing of entire genomic sequences (Fleischmann et al., 1995; Iguchi et al., 2009; NCBI genomic database, 2012). This has allowed determination of evolutionary relationships among different organisms based upon large numbers of different gene/protein sequences using a variety of approaches (Gupta, 1998; Haggerty et al., 2009; Puigbo et al., 2009; Blair and Murphy, 2011).

The comparative genomic analyses have revealed that phylogenetic relationships deducted based upon different genes and protein sequences are not congruent and lateral gene transfer (LGT) among different taxa is indicated as the main factor responsible for this lack of concordance (Gogarten et al., 2002; Bapteste and Boucher, 2008; Dagan et al., 2008; Puigbo et al., 2009; Swithers et al., 2009; Andam and Gogarten, 2011). This has led to questioning of whether the Darwinian model of evolution involving vertical inheritance of genes from parents to progenies (Darwin, 1859) is applicable to the prokaryotes (Doolittle, 1999; Pennisi, 1999; Gogarten et al., 2002; Dagan and Martin, 2006; Doolittle and Bapteste, 2007; Dagan et al., 2008; Bapteste et al., 2009; Williams et al., 2011). Multiple mechanisms are known to contribute to the evolution of an organism's genomes including genes that are acquired vertically from the parent organism, evolution of new genes by gene duplication and divergence, gain of new genes by means of LGTs, as well as gene losses in various lineages (Bapteste et al., 2009; Ragan and Beiko, 2009; Treangen and Rocha, 2011; Williams et al., 2011). LGT, in particular, is being increasingly thought to have an overbearing influence on prokaryotic genome composition. Although rRNAs, ribosomal proteins and other genes involved in the information transfer processes are considered less prone to LGTs due to their involvement in complex gene networks (Jain et al., 1999; Sorek et al., 2007), recent studies indicate that no single gene/protein is completely immune to this process (Yap et al., 1999; Doolittle and Bapteste, 2007; Dagan et al., 2008). Some recent studies have estimated that over time most genes (81 ± 15%) have undergone at least one LGT event (Doolittle, 1999; Dagan and Martin, 2007; Doolittle and Bapteste, 2007; Dagan et al., 2008). These studies in large part form the basis of the hypothesis that LGTs have led to abolishment of all signals that can be used for determination of prokaryotic evolutionary relationships and a call for uprooting the tree of life (Martin, 1999; Pennisi, 1999; Doolittle, 2000; Gogarten et al., 2002; Delsuc et al., 2005; Bapteste et al., 2009).

Although the importance of LGTs in genome evolution is widely accepted, there is considerable disagreement concerning the prevalence of LGTs and their impact on prokaryotic evolutionary relationships. While some authors have indicated that LGT is so profuse that its influence disguises the Darwinian mode of evolution involving vertical inheritance of genes (Gogarten et al., 2002; Bapteste et al., 2005b, 2009; Doolittle and Bapteste, 2007; Koonin, 2007), others have inferred that the incidences of LGTs are either very minimal or limited and those genes that are laterally transferred have little impact on prokaryotic phylogeny (Wolf et al., 2002; Kurland et al., 2003; Dutilh et al., 2004; Beiko et al., 2005; Kunin et al., 2005; Kurland, 2005; Galtier, 2007; Puigbo et al., 2009; Gao and Gupta, 2012a). However, there are no standardized methods to assess LGTs and the methods used to infer LGTs are varied and based upon large numbers of often poorly supported assumptions (Koski and Golding, 2001; Koski et al., 2001; Ragan, 2001; Beiko et al., 2005; Boto, 2010). Thus, the prevalence of LGTs differ greatly among different studies and often similar datasets have led to dissimilar conclusions (Koski et al., 2001; Ragan, 2001; Wang, 2001; Lerat et al., 2003; Susko et al., 2006; Zhaxybayeva et al., 2007; Marri and Golding, 2008; Roettger et al., 2009). Therefore, prior to concluding that in view of LGTs the Darwinian mode of evolution is not a suitable model for prokaryotes, reliability of the incidences of LGTs and their overall impact on the evolutionary relationships should be critically examined.

Despite the prevalence of LGTs, phylogenetic trees based upon 16S rRNA as well as numerous single genes as well multi-gene analyses strongly support the existence of large numbers of distinct phyla of bacteria (Ludwig and Klenk, 2005). Additionally, these trees also clearly delineate many discrete taxonomic clades within these phyla (Woese, 1987; Ludwig and Klenk, 2005; Ciccarelli et al., 2006; Wu et al., 2009; Gao and Gupta, 2012a). In a recent detailed study Puigbo et al. (2009) reported construction of phylogenetic trees for 6901 prokaryotic genes. Although there were significant topological differences among these trees, a consistent phylogenetic signal was observed in most of these trees, indicating that the LGT events, which were of random nature, did not obscure the central trend resulting from the vertical transfer of genes. The fact that similar prokaryotic clades at different taxonomic levels (ranging from phyla to genera) are consistently identified in phylogenetic trees based upon different gene/protein sequences strongly indicates that the distinctness of the prokaryotic taxa and their evolutionary relationships are in large part discernible and they have not been obliterated by LGTs (Woese, 1987; Daubin et al., 2002; Kurland et al., 2003; Lerat et al., 2003; Beiko et al., 2005; Kurland, 2005; Ludwig and Klenk, 2005; Ciccarelli et al., 2006; Ragan and Beiko, 2009; Wu et al., 2009; Boto, 2010; Yarza et al., 2010; Gupta, 2010b; Gao and Gupta, 2012a). To account for the above observations and the occurrences of LGTs, it has been suggested that the prokaryotic evolution has both tree-like (at intermediate phylogenetic depths) and non-tree (or net-like) (at the base and tips) characteristics (Dagan et al., 2008; Puigbo et al., 2009, 2010; Swithers et al., 2009; Boto, 2010; Beiko, 2011; Dagan, 2011; Kloesges et al., 2011; Popa et al., 2011).

The availability of genome sequences is also enabling development of novel and independent sequence based approaches for determining the evolutionary relationships among organisms and to assess the impact of LGTs on these relationships. In this review, we provide a summary of our recent work in this area based upon two different types of molecular markers that we have used successfully for understanding the evolutionary relationships among prokaryotes. Based upon these markers it is now possible to identify different prokaryotic taxa ranging from phyla to genera in clear molecular terms and the evolutionary relationships among them can also be reliably deducted (Gupta and Griffiths, 2002; Gupta, 2009, 2010a; Gao and Gupta, 2012b). The relationships revealed by these new approaches strongly support a tree-like branching pattern among prokaryotes and the observed incidences of LGTs, which exhibit no specific pattern or statistical significance, apparently have no major impact on the derived relationships. It is contended that these molecular markers provide valuable means for developing a reliable phylogeny and taxonomy of the prokaryotic organisms.

## Usefulness of conserved signature indels (CSIs) and conserved signature proteins (CSPs) for understanding evolutionary relationships among prokaryotes

Of the two kinds of molecular markers that we are using for studying prokaryotic evolution, the *c*onserved *s*ignature *i*ndels (inserts or deletions), or CSIs, in protein sequences comprises an important category (Gupta, 1998, 2010a; Griffiths and Gupta, 2001). The CSIs that provide useful molecular markers for evolutionary studies are generally of the same lengths and they are flanked on both sides by conserved regions to ensure that the observed changes are not caused by alignment artifacts (Gupta, 1998; Gupta and Griffiths, 2002; Jordan and Goldman, 2012). When such CSIs are present in the same position in a given protein in a group of related species, their presence is most parsimoniously explained by postulating that the genetic change leading to the CSI occurred in a common ancestor of this group and then this gene with the indel was vertically transmitted to its progeny (Rivera and Lake, 1992; Baldauf and Palmer, 1993; Gupta, 1998, 2000b; Rokas and Holland, 2000; Cutino-Jimenez et al., 2010). The CSIs that are uniquely shared by organisms of one taxa provide molecular tools for identifying the species from this taxa and consolidating the relationships among bacteria of that taxa by delimiting it in molecular terms (Gupta, 2004). Additionally, depending upon the presence or absence of a given CSI in the outgroup species, it can be determined whether the indel represents an insert or a deletion and based upon this a rooted relationship among the species of interest can be derived. Our earlier work in this regard has led to identification of large numbers of CSIs that are specific for different groups of microbes at various phylogenetic levels (Table 1; Gupta and Griffiths, 2006; Gupta, 2009; Gupta and Bhandari, 2011; Gupta and Shami, 2011; Gao and Gupta, 2012b).

**Table 1:** **Overview of the CSIs and CSPs that have been identified for some major prokaryotic taxa**.

**Taxonomic group**	**Number of CSPs/CSIs**	**References**
Archaea	*Archaeal Kingdom specific*: 16 CSPs	Gao and Gupta, 2007; Gupta and Shami, 2011
	*Subgroups*: Thaumarchaeota—6 CSIs/201 CSPs, Euryarchaeota—6 CSPs, Thermoacidophiles—77 CSPs, Halophiles—127 CSPs, Methanogens—31 CSPs, Thermococcus-Pyrococcus clade—141 CSPs	
Crenarchaeota	*Phylum specific*: 6 CSIs, 13 CSPs	Gupta and Shami, 2011
	*Subgroups*: Sulfolobales—3 CSIs/151 CSPs, Thermoproteales—5 CSIs/25 CSPs, Desulfurococcales—4CSPs, Sulfolobales-Desulfurococcales clade—2 CSIs/18 CSPs	
Thaumarchaeota	>200 CSPs	Gupta and Shami, 2011
Thermotogae	*Phylum specific*: 18 CSIs	Gupta and Bhandari, 2011
	*Subgroups*: Thermotoga genus—13 CSIs, Thermosipho genus—7 CSIs, Thermosipho-Fervidobacterium clade—13 CSIs, Thermotoga-Thermosipho-Fervidobacterium clade—5 CSIs, Petrotoga-Kosmotoga clade—4 CSIs	
Cyanobacteria	*Phylum specific*: 39 CSPs/10 CSIs	Gupta, 2009; Gupta and Mathews, 2010
	*Subgroups*: Cyanobacterial Clade A—14 CSPs/1 CSI, Other Cyanobacteria (outside clade A)—5 CSPs/4 CSIs, Cyanobacterial Clade C—60 CSPs, Nostocales—65 CSPs, Chroococcales—8 CSPs, *Synechococcus*—14 CSPs, *Prochlorococcus*—19 CSPs, Low B/A type *Prochlorococcus*—67 CSPs	
Chlamydiae	*Phylum specific*: 59 CSPs/8 CSIs	Gupta and Griffiths, 2006
	*Subgroups*: Chlamydiaceae—79 CSPs, Chlamydophila—20 CSPs, Chlamydia—20 CSPs	
Bacteroidetes, chlorobi and fibrobacteres	*Phylum specific*: 1 CSP/2 CSIs	Gupta, 2004
	*Subgroup specific*: Bacteroidetes—27 CSPs/2 CSIs, Chlorobi—51 CSPs/2 CSIs, Bacteroidetes and Chlorobi clade—5 CSPs/3CSIs	
Actinobacteria	*Phylum specific*: 24 CSPs/4 CSIs	Gao and Gupta, 2005, 2012b; Gao et al., 2006
	*Subgroup specific*: CMN group—13 CSPs, *Mycobacterium* and *Nocardia*—14 CSIs, *Mycobacterium*—24 CSPs, *Micrococcineae*—24 CSPs, Corynebacteriales—4 CSPs/2 CSIs, Bifidobacteriales—14 CSPs/1 CSI	
Deinococcus-thermus	*Phylum specific*: 65 CSPs/8 CSIs	Griffiths and Gupta, 2004a, 2007a
	*Subgroup specific*: Deinococci—206 SPs	
Aquificae	*Phylum specific*: 10 CSPs/5 CSIs	Griffiths and Gupta, 2006b, 2004b
α-proteobacteria	*Class specific*: 6 CSPs/13 CSIs	Gupta and Mok, 2007
	*Subgroups*: Rickettsiales—3 CSPs/2 CSIs, Rickettsiaceae—4 CSPs/5 CSIs, Anaplasmataceae—5 CSPs/2 CSIs, Rhodobacterales-Caulobacter-Rhizobiales clade—2 CSIs, Rhodobacterales-Caulobacter clade—1 CSI, Rhizobiales—6 CSPs/1CSI, Bradyrhizobiaceae—62 CSPs/2CSIs	
γ-proteobacteria	*Class specific*: 4 CSPs/1 CSI	Gao et al., 2009
	*Subgroups*: 20 CSPs, 2 CSIs for various subgroup combinations of subgroups	
ε-proteobacteria	*Class specific*: 49 CSPs/4 CSIs	Gupta, 2006
	*Subgroups*: *Wolinella*-*Helicobacter* clade—11 CSPs/2 CSIs, *Campylobacter* genus—18 CSPs/1 CSI	
Pasteurellales	*Order specific*: 44 CSIs	Naushad and Gupta, 2012
	*Subgroups*: Pasteurellales Clade I—13 CSIs, Pasteurellales Clade II—9 CSIs	
Clostridia sensu stricto	*Genus specific*: 10 CSPs/3 CSIs	Gupta and Gao, 2009

The table provides general information regarding the number of CSIs and CSPs identified for many taxonomic groups on which genomic studies have been conducted. Further details can be obtained from the corresponding studies.

The second kind of molecular markers that we have usefully employed in our systematic and evolutionary studies are whole proteins that are uniquely found in particular groups or subgroups of bacteria (Gupta, 2006; Gupta and Griffiths, 2006; Gupta and Mok, 2007; Gao and Gupta, 2012b). Comparative analyses of genomic sequences have indicated that many conserved proteins are uniquely present in all species from particular groups, at different phylogenetic depths (Daubin and Ochman, 2004; Lerat et al., 2005; Gupta, 2006; Gupta and Griffiths, 2006; Gupta and Mok, 2007; Dutilh et al., 2008; Gao and Gupta, 2012b). Because of their unique presence in species from particular phylogenetic clades of species, it is likely that the genes for these CSPs originated once in a common ancestor of these groups and then vertically acquired by all its descendants. Because of their taxa specificity these CSPs again provide valuable molecular markers for identifying different groups of species in molecular terms and for evolutionary studies (Gao and Gupta, 2007; Gupta and Mathews, 2010; Gupta, 2010b). However, when a CSP (or CSI) is confined to certain species/strains, then based upon this information alone, it is often difficult to determine whether these species form a clade in the phylogenetic sense or not. Hence, to understand the evolutionary significance of these signatures, such studies are generally performed in conjunction with phylogenetic analysis, which provides a reference point for evaluating the significance of various CSIs and CSPs (Gao and Gupta, 2007; Gupta and Mathews, 2010; Gupta, 2010b).

Molecular markers in the form of CSIs and CSPs have proven useful for examining or consolidating prokaryotic relationships at domain, phylum as well as intra-phylum levels. Table 1 provides a summary of some bacterial and archaeal taxa for which CSIs and CSPs have been identified (Gupta, 2010a). Two recent detailed studies based upon CSIs and CSPs have focused upon understanding evolutionary relationships within the phylum Thermotogae and the domain Archaea (Gao and Gupta, 2007; Gupta and Bhandari, 2011; Gupta and Shami, 2011). To illustrate the usefulness of these molecular markers for elucidation of prokaryotic evolutionary relationships, and to assess the influence of LGTs on the derived inferences, results for these two taxonomic groups are reviewed here.

## Molecular markers for the thermotogae

The species of the phylum Thermotogae are a group of hyperthermophilic, anaerobic, gram-negative bacteria recognized by a distinctive toga-like sheath structure and their ability to grow at high temperatures (Huber et al., 1986). The approximately 90 species of this phylum are currently divided into nine Genera within a single family termed the Thermotogaceae (Euzeby, 2011; NCBI Taxonomy, 2012). The Thermotogae species, prospectively, are important tools for industrial and biotechnological applications due to the ecological niche they inhabit and the thermo-stable proteins that they harbor (Conners et al., 2006). With the publication of the genome for *T. maritima*, the first species from this phylum (Nelson et al., 1999), the Thermotogae were brought to the forefront of LGT debate. This was due to the fact that based upon Blast searches it was determined that for about 25% of the genes from *T. maritima* genome, the closest blast hits were from archaeal species rather than any bacteria, leading to the inference that Thermotogae species have incurred high degree of LGTs with the archaeal organisms (Nelson et al., 1999). Upon revisiting this issue, Zhaxybayeva et al. (2009) found that for only about 11% of the Thermotogae proteins Archaea were the closest hits, but that the Thermotogae proteins exhibited maximal similarity (42–48% of genes) to the Firmicutes. Based upon these observations, the Thermotogae species genomes were proposed to be a chimera composed of different bacterial and archaeal sources (Zhaxybayeva et al., 2009). However, these estimates for LGTs have been questioned in other studies which indicate that much less (6–7%) of the Thermotogae genome has been laterally transferred (Garcia-Vallve et al., 2000; Ochman et al., 2000). Further, in view of the fact that Thermotogae species branch in proximity of the Firmicutes phylum (Gupta, 2001; Griffiths and Gupta, 2004b), the observation that a preponderance of the top hits for the Thermotogae species are from Firmicutes is an expected results, and it does not indicate that these genes have been laterally transferred (Zhaxybayeva et al., 2009; Andam and Gogarten, 2011).

**Figure 1:**
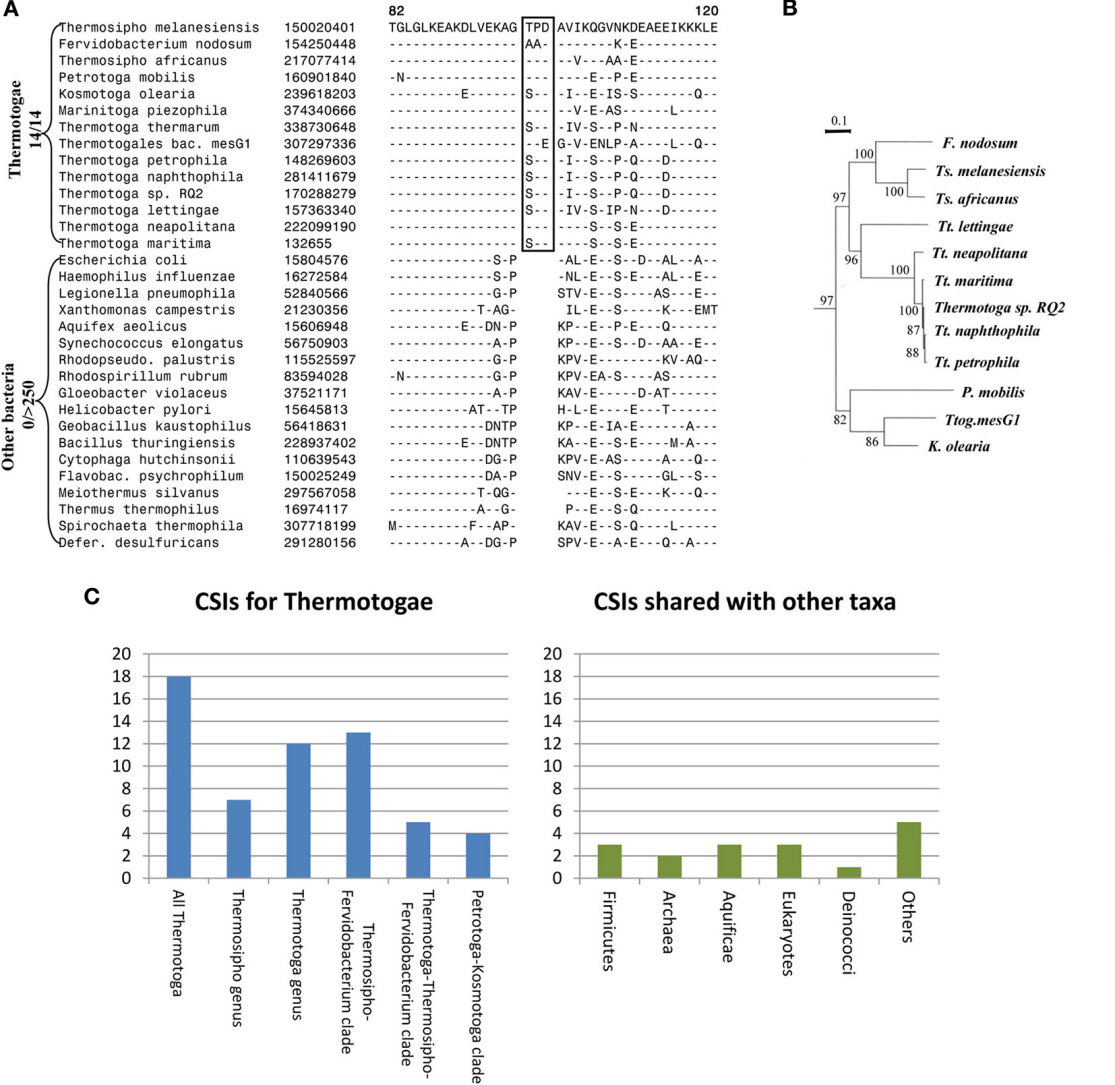
**Evolutionary relationships among Thermotogae species based upon CSIs and a Phylogenetic Tree. (A)** Partial sequence alignment for the ribosomal protein L7 showing a 3 aa CSI (boxed) that is specific for all detected species of the Thermotogae phylum. The dashes in the alignment (−) indicate amino acid identity with the corresponding residue in the top line; **(B)** A maximum likelihood tree for the 12 sequenced Thermotogae species based upon concatenated sequences for 12 conserved proteins. **(C)** A summary diagram showing the species specificities of different CSIs identified for the Thermotogae group of species. The left panel highlights the CSIs that are specific for the entire Thermotogae phylum or its sub-groups, whereas the right panel indicates the CSIs that were also present in some non-Thermotogae organisms. Figures 1A,B modified from Gupta and Bhandari (2011).

Apart from their unique protein toga, the species of the phylum Thermotogae are assigned to this group and divided into its different genera primarily on the basis of their branching in the 16S rRNA trees (Reysenbach, 2001; Huber and Hannig, 2006; Zhaxybayeva et al., 2009; Yarza et al., 2010). Until recently, no unique molecular or biochemical characteristics were known that could distinguish the species of this phylum from other bacteria. For identification of molecular markers that could possibly define this phylum and its sub-taxa, a genome wide analysis was performed on protein sequences from 12 Thermotogae spp. whose genomes were available (Gupta and Bhandari, 2011). The protein sequences from these 12 species as well as species representing other bacteria phyla were aligned and examined for the presence of CSIs that were uniquely present in Thermotogae species or those that were commonly shared with some other bacteria. The analysis identified numerous CSIs specific for all Thermotogae. An example of a CSI consisting of a 3 aa long insert in the ribosomal protein L7 that is exclusively present in all sequenced Thermotogae species, including two recently sequenced species, is shown in Figure 1A. The unique presence of this CSI of the same length, at the same position in this universally distributed protein, in different species from the phylum Thermotogae indicates that the genetic change leading to this CSI occurred once in the common ancestor of the Thermotogae species. In addition to this CSI, this study also identified 17 other CSIs in other important proteins such as DNA recombination protein RecA, DNA polymerase I and tryptophanyl-tRNA synthetase that are also specific for the species from the phylum Thermotogae (Gupta and Bhandari, 2011).

In addition to the large numbers of CSIs that were uniquely present in all Thermotogae species, this study also identified many CSIs that were specific for different sub-groups within the phylum Thermotogae (Gupta and Bhandari, 2011). These included 13 CSIs that were specific for the species of the genus *Thermotoga* and seven others that distinguished species of the genus *Thermosipho* from all others. However, it was observed that the species *Thermotoga lettingae* shared only 1 of 13 CSIs that were otherwise commonly present in other species of this genus. This suggests that *T. lettingae*, which is distantly related to all other *Thermotoga* species, should be assigned to a separate genus. Besides these CSIs that were specific for the species of these two genera, 13 CSIs supported a specific relationships among species of the *Fervidobacterium* and *Thermosipho* genera; 5 CSIs were shared by species from the genus *Thermotoga* and those from the *Fervidobacterium-Thermosipho* clade; and 4 CSIs supported a grouping of the *Petrotoga* and *Kosmotoga* genera along with the species *Thermotogales bacterium MesG1.Ag.4.2* (Figure 1C, left panel; Gupta and Bhandari, 2011). Importantly, all of the relationships indicated by various CSIs were also independently observed in a phylogenetic tree for the Thermotogae species based upon concatenated sequences for 12 conserved proteins (Figure 1B).

The CSIs identified in the above study independently and strongly supported different nodes observed in the phylogenetic tree for Thermotogae species all the way from phylum to genus level. If the hypothesis that LGT events have abolished the ability to discern prokaryotic relationships was correct, then it should have been difficult to identify discrete molecular markers supporting distant relationships among these species. At the very least, the Thermotogae species would have shown relationships with species of other prokaryotic groups such as Firmicutes or Archaea as frequently as they did with one another. In this study, in addition to the CSIs that were specific for the Thermotogae species (Figure 1C, left panel), several CSIs were also identified that the Thermotogae shared with species from other prokaryotic or eukaryotic organisms (Figure 1C, right panel). However, such CSIs, suggesting possible LGT between Thermotogae and other taxa, were far outweighed by CSIs supporting the monophyletic, tree-like relationships among the species of the phylum (left panel) (Gupta and Bhandari, 2011). Assuming that all the CSIs that the Thermotogae shared with other groups are due to LGT, less than 20% (16 of 85) of all Thermotogae genes containing these CSIs have incurred LGTs (Gupta and Bhandari, 2011). Moreover, these presumed LGT events are of random nature and in no case do the Thermotogae species share more than a total of 3 CSIs with any particular phyla of species. Additionally, in most of these cases only a few species from these other taxa contained the indels that were present in most or all Thermotogae species (Gupta and Bhandari, 2011). Thus, these other CSIs, although they are present in a few isolated species from other taxa, are also largely specific for the Thermotogae species and they do not affect the ability of other CSIs to clearly discriminate Thermotogae species from all other bacteria or to deduce the evolutionary relationships amongst species from this phylum.

The shared presence of similar CSI in unrelated taxa can result from two different possibilities, either the gene with the CSI was laterally transferred among the two groups or that independent CSIs owing to two separate genetic events are responsible for these CSIs. After identification of such CSIs, tree-making approaches can be used to test if the presence of the indel in the two groups is due to LGT. Previously, in our work, a number of CSIs in the GlyA and MurA proteins that were commonly shared by the Chlamydiae and a subgroup of Actinobacteria were shown to be due to lateral transfer of genes from Actinobacteria to a common ancestor of the Chlamydiae (Griffiths and Gupta, 2006a). Recently, the shared presence of several CSIs in the bacterio-chlorophyll biosynthesis proteins by unrelated phyla of photosynthetic prokaryotes has also been shown to be due to LGTs (Raymond et al., 2002; Gupta, 2012). However, in many other instances phylogenetic analyses have not supported LGT as the possible reason for the presence of a related CSI in unrelated taxa. In these cases, similar CSIs have originated independently in these lineages due to their presumed similar functions in these particular taxa.

## Molecular markers for the archaea and its sub-groups

Archaea are widely recognized as the third domain of life. They generally inhabit extreme environments such as those of extreme temperature, pH or salinity, where little to no other life exists (Woese et al., 1990). However, recent studies indicate that archaeal species are widespread in the environment and they play a major role in the carbon and nitrogen cycles (Pace, 1997; Herndl et al., 2005; Leininger et al., 2006). Some archaeal species have been found to be commensal organisms residing in human colons (Oxley et al., 2010). The Archaea are generally divided into two main phyla, the Crenarchaeota and Euryarchaeota, based on 16S rRNA data and other phylogenetic data (Woese et al., 1990; Gribaldo and Brochier-Armanet, 2006). The Crenarchaeotes are described as thermophiles with sulfur-reducing capabilities while the Euryarchaeotes are metabolically and morphologically quite diverse (Gribaldo and Brochier-Armanet, 2006; Gupta and Shami, 2011). The mesophilic Crenarchaeota have been recently placed into a separate phylum called the Thaumarchaeota (Brochier-Armanet et al., 2008; Gupta and Shami, 2011).

**Figure 2:**
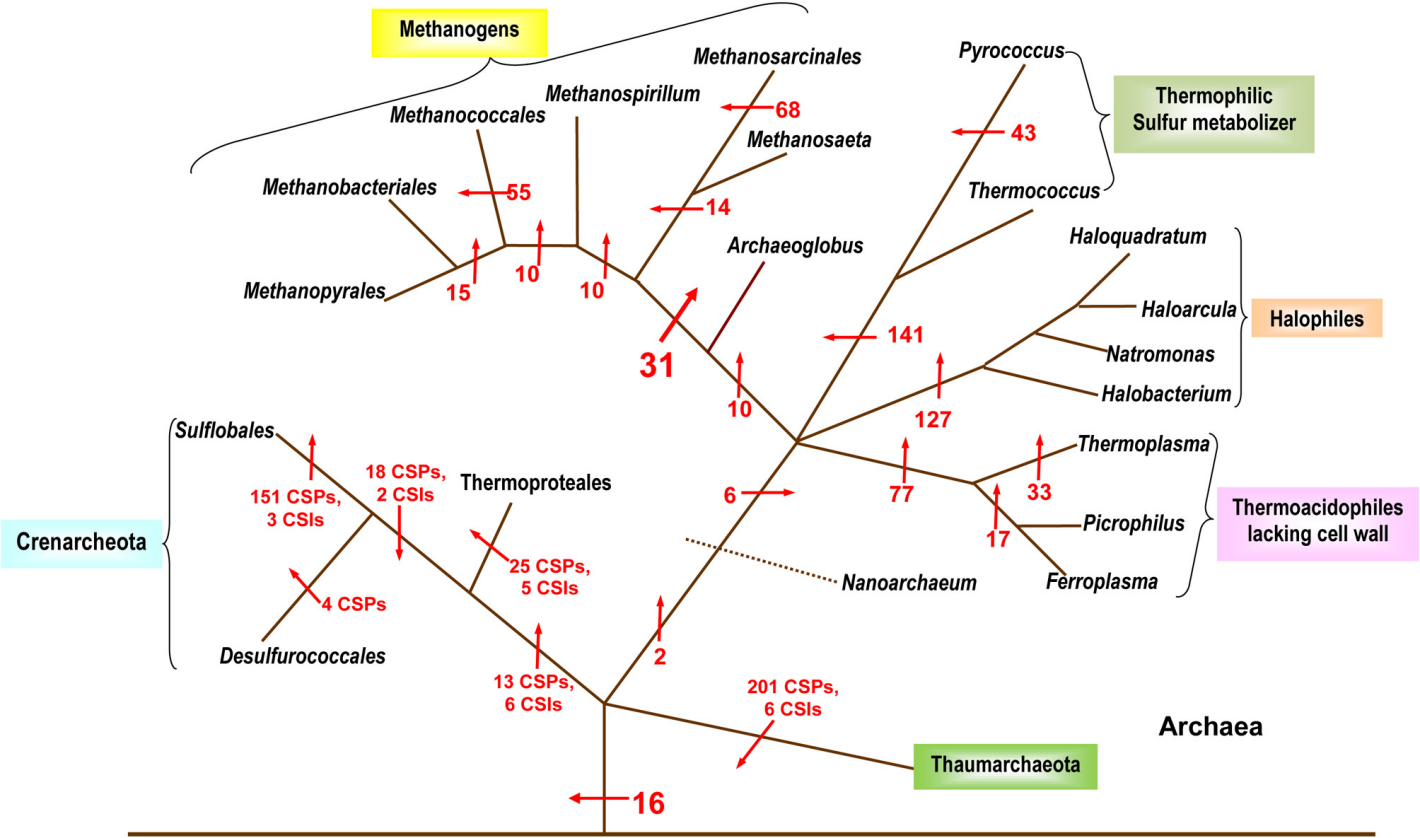
**A summary diagram showing the various molecular markers that have been identified for the Archaeal kingdom and its subgroups.** The arrows indicate the suggested evolutionary stages where the proteins unique for a particular taxa are proposed to have been introduced. The numbers beside the arrows indicate the number of CSIs and CSPs specific for the various taxa (these numbers indicate CSPs unless otherwise noted). The branching pattern shown is based solely upon the distribution patterns of CSPs and CSIs. Modified from Gao and Gupta (2007) and Gupta and Shami (2011).

Despite the importance of Archaea in different environments and in understanding of the evolutionary history of life on earth (Woese et al., 1990; Gupta, 2000a), until recently, very few molecular characteristics were known that are uniquely shared by all Archaea. Additionally, as the higher taxonomic groups within Archaea are described primarily based upon 16S rRNA trees, the characteristics that are unique to different phyla, classes, orders and families of the Archaea have scarcely been elucidated (Boone et al., 2001). The utilization of archaeal genomes for discovery of CSPs as well as CSIs has provided significant information in the form of molecular markers that are distinctive characteristics of Archaea and its taxonomic sub-groups. In 2007, a comprehensive analysis was performed on available archaeal genomes to search for CSPs that were unique to either all Archaea or many of its sub-groups (Gao and Gupta, 2007). Over 1400 such proteins distinctive of Archaea or its main taxa were discovered (Figure 2). In the analysis, sixteen proteins specific to all or most Archaea were identified that were not present in any bacterial or eukaryotic organism. Numerous proteins whose homologs were limited to the Crenarchaeota, Euryarchaeota and other sub-groups such as the Thermococci, Thermoplasmata, and Halobacteriales were also detected (Figure 2). Significantly, this study also identified 31 proteins that were commonly shared by all methanogenic bacteria (Gao and Gupta, 2007). In the 16S rRNA and other phylogenetic trees, the methanogenic Archaea do not form a monophyletic lineage, but instead are split into a number of distinct clusters separated by non-methanogenic Archaea (Burggraf et al., 1991; Brochier et al., 2004; Bapteste et al., 2005a; Gao and Gupta, 2007). Because most of the proteins that are commonly shared by various methanogens are generally involved in functions related to methanogenesis and their genes are clustered into a few large operons in genomes (Harms et al., 1995; Tersteegen and Hedderich, 1999; Grabarse et al., 2001; Gao and Gupta, 2007), it is likely that the genes for these proteins have been laterally acquired by different Archaea. This could provide a plausible explanation for the observed discrepancy in the branching of methanogenic Archaea in phylogenetic trees and their unique sharing of genes for these proteins (Gao and Gupta, 2007).

A recent analysis has further added to the catalogue of molecular signatures for the archaeal organisms (Gupta and Shami, 2011). The focus of this study was on identifying CSIs and CSPs that were specific for the Crenarchaeota and Thaumarchaeota phyla (Gupta and Shami, 2011). Six CSIs and 13 CSPs specific for all species of the phylum Crenarchaeota were identified along with numerous markers for its different orders: the Sulfolobales (151 CSPs, 3 CSIs), Thermoproteales (25 CSPs, 5 CSIs) and the Desulfurococcales (4 CSPs). The study also described the markers (18 CSPs and 2 CSIs) indicative of a close relationship among the Sulfolobales and the Desulfurococcales. The discriminative ability of CSPs is highlighted by the results of blast searches on some CSPs that are specific for the Crenarchaeota or its main groups (Sulfolobales, Thermoproteales, Desulfurococcales and Acidilobales) that are shown in Table 2. In these cases, BLASTP searches were carried out on these proteins and the results for all species for whom the observed *E*-values were significant are shown. From the results presented in Table 2, it is evident that the first 2 CSPs are specific for the Crenarchaeota phylum, the next two are uniquely found in various species belonging to the orders Desulfurococcales, Acidilobales and Sulfolobales, whereas the last 5 CSPs are distinctive characteristics of species belonging to either the Desulfurococcales (and Acidilobales), the Sulfolobales, or the Thermoproteales orders.

**Table 2:** **A series of proteins specific for the Crenarchaeota and its sub-groups**.

	**Protein accession #**	**NP_147640**	**NP_147284**	**BAA81469**	**NP_147588**	**YP_001041009**	**YP_254810**	**YP_254922**	**NP_559041**	**NP_559897**
	**Protein length**	**262 aa**	**143 aa**	**98 aa**	**228 aa**	**127 aa**	**228 aa**	**270 aa**	**626 aa**	**113 aa**
**Desulfurococcales**	*Aeropyrum pernix*	0.0	9e–98	5e–64	7e–161	7e–22	–	–	–	–
	*Hyperthermus butylicus*	3e–46	9e–43	1e–20	1e–23	3e–25	–	–	–	–
	*Ignicoccus hospitalis*	3e–41	–	5e–27	4e–19	3e–25	–	–	–	–
	*Desulfurococcus kamchatkensis*	7e–46	1e–21	2e–20	5e–17	7e–32	–	–	–	–
	*Staphylothermus marinus*	4e–56	1e–25	3e–21	3e–21	2e–85	–	–	–	–
**Acidilobales**	*Acidilobus saccharovorans*	9e–56	4e–36	4e–21	1e–46	1e–19	–	–	–	–
**Sulfolobales**	*Sulfolobus tokodaii*	4e–40	2e–29	3e–20	7e–26	–	1e–77	1e–80	–	–
	*Sulfolobus islandicus*	4e–42	6e–30	1e–25	1e–15	–	7e–50	8e–65	–	–
	*Sulfolobus acidocaldarius*	7e–34	3e–23	4e–22	4e–24	–	2e–162	0.0	–	–
	*Sulfolobus solfataricus*	1e–41	7e–30	5e–26	8e–15	–	5e–50	8e–64	–	–
	*Metallosphaera sedula*	3e–31	3e–33	3e–20	1e–22	–	4e–39	8e–60	–	–
**Thermoproteales**	*Pyrobaculum aerophilum*	9e–18	3e–11	–	–	–	–	–	0.0	2e–73
	*Pyrobaculum islandicum*	3e–18	3e–11	–	–	–	–	–	0.0	6e–54
	*Pyrobaculum arsenaticum*	1e–18	1e–10	–	–	–	–	–	0.0	2e–63
	*Pyrobaculum caldifontis*	6e–22	7e–11	–	–	–	–	–	0.0	1e–60
	*Thermofilum pendens*	1e–35	5e–30	–	–	–	–	–	1e–42	3e–10
	*Caldivirga maquilingensis*	1e–17	4e–8	–	–	–	–	–	1e–87	2e–22
	*Thermoproteus neutrophilus*	2e–19	7e–11	–	–	–	–	–	0.0	5e–61
	*Thermoproteus tenax*	3e–15	6e–10	–	–	–	–	–	0.0	4e–46
**Top non-Crenarchaeota hit**		*Brucella melitensis* (2e–1)	*Desulfobacterium autotrophicum* (8e–1)	*Aromatoleum aromaticum* (4e–1)	*Serpula lacrymans* (7e–1)	*Clonorchis sinensis* (3e–1)	*Granulicatella elegans* (6e–1)	*Encephalitozoon cuniculi* (7e–1)	*Burkholderia cenocepacia* (9e–1)	*Sordaria macrospora* (1e–1)

Blastp searches were carried out on proteins specific for the Crenarchaeota or its sub-groups and the results for representative species from different sub-groups of the Crenarchaeota are shown with the observed *E*-values. *E*-values greater than 1e–3 are considered insignificant hits with lack of homology to the query protein sequence.

The dashes (–) indicate that the homolog for the protein query was not detected in the BlastP searches.

Top non-Crenarchaeota hits indicate detection of species outside the Crenarchaeota that were observed to have the lowest *E*-value scores.

In this study, more than 200 CSPs for various members of the newly defined Thaumarchaeota phylum were also identified (Gupta and Shami, 2011). The Thaumarchaeota are composed of several organisms previously included in the Crenarchaeota (Brochier-Armanet et al., 2008). The two phyla appear as sister groups in phylogenetic analysis and they also share 3 CSIs and 10 CSPs with each other (Gupta and Shami, 2011). Nevertheless, the two groups can be phylogenetically differentiated and numerous markers have been identified for each group that helps to define them molecularly as individual taxa (Gupta and Shami, 2011). A summary diagram depicting the various molecular markers specific for the archaeal species is shown in Figure 2. It should be noted that CSIs were only identified for the Thaumarchaeota and the Crenarchaeota and no detailed analysis to identify CSIs has thus far been carried out on the Euryarchaeota.

The two studies noted above have identified numerous CSIs and CSPs for the Archaea, its main phyla (Euryarchaeota, Crenarchaeota, Thaumarchaeota) and a number of its sub-phylum level taxa (Sulfolobales, Thermococcales, Halobacteriales, etc.; Gao and Gupta, 2007; Gupta and Shami, 2011). Except for the methanogens, the distribution patterns of the identified CSIs and CSPs are also strongly supported by the phylogenetic branching pattern of the archaeal organisms (Gribaldo and Brochier-Armanet, 2006; Gao and Gupta, 2007; Brochier-Armanet et al., 2008; Gupta and Shami, 2011). Considering the specificities of these molecular markers for either all Archaea or different clades of Archaea, these results strongly indicate that LGTs have not obliterated the phylogenetic signal necessary to delineate the evolutionary relationships among this domain of prokaryotes. The discovered CSIs and CSPs also provide novel tools for the identification of different groups of Archaea in various environments.

## The usefulness of the CSIs for understanding bacterial phylogeny and taxonomy

**Figure 3:**
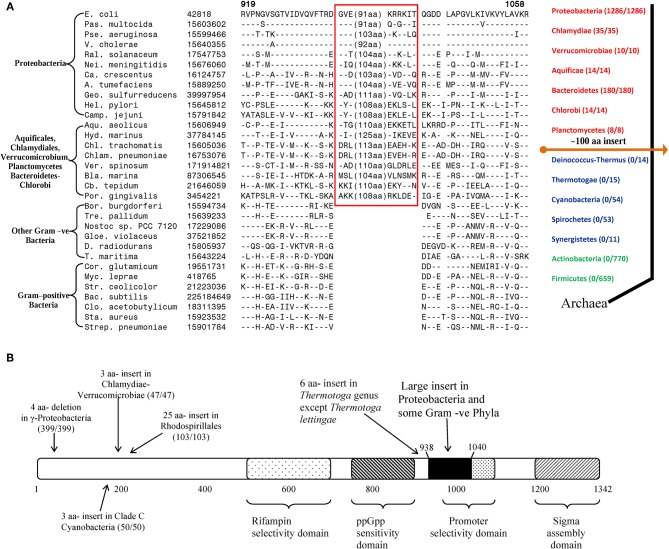
**Evolutionary significance of various identified CSIs in the RNA polymerase β subunit. (A)** A portion of the RpoB sequence alignment showing a large insert (boxed) that is distinctive characteristic of all Proteobacteria and some Gram-negative phyla (Chlamydiae-Verrucomicrobiae, Aquificales, Planctomycetes, and Bacteroidetes-Chlorobi), but not found in other phyla of bacteria. Due to the large size of the insert, its entire sequence is not shown. Dashes (–) indicate identity with the amino acid on the top line. On the right is a linear representation of prokaryotic relationships based on the presence and absence of this CSI. The numbers in the brackets indicate the species of each phylum, which have been identified to contain the CSI. **(B)** A schematic representation of the sequence for *E. coli* RNA polymerase β subunit (RpoB) showing some functionally important regions and the positions of different lineage-specific inserts that have been identified within this protein. The large insert depicted in **(A)** (≈ 100 aa in *E. coli)* is shown in solid black. The positions of CSIs for different groups are roughly indicated using arrows. The values in the brackets identify the number of organisms in each respective group and the number of these species to harbour the indicated CSI. In all cases no organism outside of the indicated group was identified to contain the indel. The indicated CSIs have been described in earlier work (Griffiths and Gupta, 2004b, 2007b; Gupta and Mok, 2007; Gao et al., 2009; Gupta and Bhandari, 2011; Naushad and Gupta, 2012).

In addition to the CSIs that are specific for particular prokaryotic taxa, several of the identified CSIs have also proven useful in clarifying the branching order and interrelationships amongst different bacterial phyla (Gupta, 2001, 2011; Gupta and Griffiths, 2002). One example of these kinds of CSIs, which are referred to as the main-line signatures in our work, is shown in Figure 3A. In this case, a large ~100 aa insert in the β subunit of RNA polymerase protein (RpoB) is commonly shared by all of the sequenced species belonging to the phyla Proteobacteria (different subclasses), Aquificae, Chlamydiae, Verrucomicrobiae, Bacteroidetes-Chlorobi, and Planctomycetes (Griffiths and Gupta, 2007b). This insert is present in all of the >1500 sequences that are available from species from these phyla. On the other hand, this CSI is not found in any of the >1500 sequences available from various species belonging to the phyla Firmicutes, Actinobacteria, Chloroflexi, Cyanobacteria, Deinococcus-Thermus, Synergistetes, Spirochaetes, etc. This insert is also not found in the archaeal RpoB homologs, thus providing evidence that this indel is an insert in the groups of species where it is found (Griffiths and Gupta, 2004b). Based upon its highly specific species distribution pattern, which argues strongly against the lateral transfer of this gene amongst various phyla, the genetic change responsible for this CSI most likely occurred in a common ancestor of the group of species that contain this CSI, after the divergence of other bacterial phyla that lack this indel as indicated in Figure 3A (right panel). A number of other main-line CSIs, which based upon their species distribution patterns have occurred at other important branch points in prokaryotic evolution, have been described in our earlier works (Griffiths and Gupta, 2001, 2004b; Gupta and Griffiths, 2002). Based upon these CSIs, it is possible to determine the branching order of most of the bacterial phyla (Gupta, 1998, 2001, 2003; Griffiths and Gupta, 2004b; see also www.bacterialphylogeny.info).

Within the highly conserved RpoB protein, in addition to the large CSI that is commonly shared by a number of bacterial phyla, several other CSIs have been identified that are specific for different groups/phyla of bacteria. The taxon specificities of these CSIs and their positions within in the RpoB polypeptide are shown in Figure 3B. These CSIs include a 4 aa deletion that is commonly and uniquely shared by a number of different orders of the γ-proteobacteria (399/399 species), a 3 aa insert that is specifically present in all of the Chlamydiae-Verrucomicrobiae species (47/47), another 3 aa insert that is a distinctive property of the Clade C cyanobacteria (50/50; Gupta, 2009), a 25 aa insert in various species from the order Rhodospirillales (103/103) and a 6 aa insert in all species from the genus *Thermotoga* except *T. lettingae* (Gupta and Griffiths, 2006; Gupta and Mok, 2007; Griffiths and Gupta, 2007b; Gao et al., 2009; Gupta and Bhandari, 2011). It is highly significant that within a single gene/protein multiple highly specific CSIs are present, each of which is specific for a different group of bacteria and help distinguish these groups from all other bacteria. These CSIs are not present in any species outside of the indicated taxa. The presence of these different taxa-specific characteristics in a single gene/protein strongly indicates that the genetic changes responsible for these CSIs occurred in the gene for this key protein at different stages in the evolution of bacterial domain and that no LGT of the gene for the RpoB protein has occurred among these taxa. Similar to the RpoB protein, multiple CSIs that are specific for different groups of prokaryotes have also been identified in many other important genes/proteins. These observations indicate that strong and consistent phylogenetic signals that are very likely not affected to any significant extent by the LGTs are still present in many conserved and universally distributed genes/proteins and these can be used to trace the evolutionary relationships among prokaryotes.

It is important to point out that virtually all of the higher taxonomic clades (above the Genus rank) within prokaryotes are currently identified solely on the basis of their branching in the 16S rRNA trees. Because the phylogenetic trees are a continuum, based upon them it has proven difficult to clearly define or delimit the boundaries of different taxonomic groups. Additionally, for virtually all of the higher prokaryotic taxa, no molecular, biochemical or physiological characteristics are known that are unique to them. Hence, a very important aspect of microbiology that needs to be understood is that in what respects do species from different main groups of bacteria differ from each other and what, if any, unique molecular, biochemical, structural or physiological characteristics are commonly shared by species from different groups? In this context, the large numbers of CSIs and CSPs for different taxonomic clades of bacteria that are being discovered by comparative genomic analyses provide novel and valuable tools for taxonomic, diagnostic, and biochemical studies (Gupta and Bhandari, 2011; Gao and Gupta, 2012b). In view of the specificities of the discovered CSIs and CSPs for different groups of prokaryotes and their retention by all species from these groups of prokaryotes, it is highly likely that these CSIs and CSPs are involved in functions that are essential for prokaryotes (Galperin and Koonin, 2004; Fang et al., 2005; Singh and Gupta, 2009; Schoeffler et al., 2010). Indeed, recent work on several CSIs have shown that they are essential for the group of organisms where they are found and the deletion or substantial changes in them led to failure of cell growth (Singh and Gupta, 2009; Schoeffler et al., 2010). Hence, further studies on understanding the cellular functions of the different taxa-specific CSIs and CSPs could lead to identification of novel biochemical and other functional characteristics that are specific for these groups of organisms.

It should also be noted that the identified CSIs and CSPs generally constitute robust molecular characteristics that exhibit high degree of predictive ability. Many of these CSIs and CSPs were discovered when the sequence information was available for very few prokaryotic species. However, despite the large increase in the number of sequenced genomes, most of these CSIs and CSPs are still specific for the originally indicated groups of prokaryotes (Gupta, 2009, 2011; Gao and Gupta, 2012b). Additionally, for several Chlamydiae-, Aquificae-, Deinococcus-Thermus- and Actinobacteria- specific degenerate primers based on conserved flanking sequences have been designed and they have been used to amplify the sequence regions predicted to contain the CSIs from large numbers of organisms for whom no sequences were available (Griffiths and Gupta, 2004a,b; Gao and Gupta, 2005; Griffiths et al., 2005). In these studies, in almost all cases the expected inserts or deletions were found to be present in previously un-sequenced organisms from the indicated groups, thus providing evidence that these CSIs and CSPs provide powerful new tools for identification of both known as well as novel species from different groups of prokaryotes.

## Conclusions

There is considerable debate at present concerning the impact of LGTs on understanding prokaryotic phylogeny. While there is little dispute that LGT plays an important role in microbial evolution, the extreme view taken by some that LGTs are so rampant within the prokaryotes that it totally masks the evolutionary signal from vertical transfer of genes (Doolittle, 2000; Gogarten et al., 2002; Doolittle and Bapteste, 2007; Dagan et al., 2008; Bapteste et al., 2009) is not supported by available evidence. As reviewed here, in phylogenetic trees based upon most gene/protein sequences all of the major groups within prokaryotes (from phylum down to genus level) are generally clearly identified, thus indicating that a strong phylogenetic signal emanating from vertical transfer of genes is maintained throughout prokaryotic evolution (Gupta, 1998, 2000b; Dutilh et al., 2004; Ludwig and Klenk, 2005; Ciccarelli et al., 2006; Puigbo et al., 2009). Most of the differences seen amongst these trees are either at the tips (i.e., species/strains levels) or at the base, i.e., relationships among the higher taxonomic clades such as phyla, class, etc. A recent study indicates that the incidence of LGTs shows linear correlation with the genome sequence and the GC content similarities of the donor and recipient organisms (Kloesges et al., 2011). Hence, while many of the observed inconsistencies between different gene trees at the species/strain levels could be due to LGTs (Puigbo et al., 2009; Kloesges et al., 2011), the differences in branching pattern at the higher taxonomic levels are perhaps in large parts due to loss of the phylogenetic signal and the lack of resolving power of the tree-based phylogenetic approaches (Gupta, 1998; Ludwig and Klenk, 2005; Puigbo et al., 2009).

In this review we have discussed the usefulness of CSIs and CSPs, as novel and important class of molecular markers for understanding the evolutionary relationships among prokaryotes. We have presented compelling evidence that based upon the species distribution patterns of these molecular signatures different prokaryotic taxa from phylum down to the genus levels can be clearly identified. Additionally, based upon these markers it is also possible to reliably deduct the evolutionary relationships amongst different prokaryotic taxa, both within a phylum and among different phyla. The evolutionary relationships deduced based upon these molecular markers generally exhibit high degree of congruency with those indicated by 16S rRNA trees or other gene/protein sequences. The analyses based upon these markers have also been able to clarify some relationships that are not resolved in phylogenetic trees. The species distribution patterns of these markers thus provide strong evidence that different clades of bacteria have evolved in a tree-like manner and that the prokaryotic organisms are not an exception to the Darwinian model of evolution. The relatively small numbers of these CSIs where the indel is also present in some unrelated species, which could be due to LGTs, show no specific pattern or relationship, thus they have minimal or no impact on the strong and consistent tree-like branching pattern that is evident from all other identified CSIs. However, it should be acknowledged that all of the work using CSIs and CSPs on understanding the evolutionary relationships among prokaryotes has thus far been carried out at genus level or higher taxa. Hence, it remains to be seen whether this approach will prove equally useful in clarifying the evolutionary relationships at the species or strain levels or not, where the evolutionary flux and the incidences of LGTs are deemed to be the highest (Daubin et al., 2003; Lerat et al., 2003; Dagan et al., 2008; Puigbo et al., 2009; Kloesges et al., 2011).

The molecular markers such as those described here in addition to their usefulness for understanding prokaryotic phylogeny also provide valuable means to address/clarify a number of important aspects of microbiology. (1) Based upon these markers different prokaryotic taxa can now be identified in clear molecular terms rather than only as phylogenetic entities. (2) Based upon them the boundaries of different taxonomic clades can also be more clearly defined. (3) Due to their high degree of specificity and predictive ability, they provide important diagnostic tools for identifying both known and unknown species belonging to these groups of bacteria. (4) The shared presence of these CSIs by unrelated groups of bacteria provides potential means for identifying novel cases of LGTs. (5) Functional studies on these molecular markers should help in the discovery of novel biochemical or physiological properties that are distinctive characteristics of different groups of prokaryotes.

Lastly, it should be acknowledged that the number of genes which harbor rare genetic changes such as these CSIs is generally small in comparison to the total number of genes that are present in any genome. However, the genes containing these CSIs are involved in different essential functions and they are often are amongst the most conserved proteins found in various organisms. Although, the criticism could be levied that the inferences based upon small numbers of genes/proteins containing these CSIs are not representative of the entire genomes (Dagan and Martin, 2006; Bapteste and Boucher, 2008), it should be emphasized that in a number of studies such as those discussed here, the reported CSIs or CSPs represent analyses of the entire genomes. Based upon these CSIs and/or CSPs, no other significant or consistent relationships or patterns among these organisms, other than those indicated here, can be derived from consideration of all of the gene/protein sequences in these genomes using these approaches. In this context it is also helpful to remember that molecular sequences like all other fossils change and disintegrate over long evolutionary periods of time and they lose their information content at different rates. Hence, a well-preserved fossil is generally considered to be far more informative than hundreds or even thousands of disintegrated fossils. Following this analogy, it is expected that not all genes/proteins will prove equally useful for understanding the evolutionary history of prokaryotes, which spans >3.5 billion years. Thus, the best we can hope for is to find significant numbers of conserved genes/proteins, which contain consistent and reliable signals such as those described in the present work, whose inferences are generally consistent with all/most other available information.

### Conflict of interest statement

The authors declare that the research was conducted in the absence of any commercial or financial relationships that could be construed as a potential conflict of interest.
